# “The Least Sacrifice of Parts,” as a Leading Principle of Surgical Practice*Read in part at the Medical Society of London, January 11th, 1875.

**Published:** 1875-05-01

**Authors:** Thomas Bryant


### "THE LEAST SACRIFICE OF PARTS" AS A LEADING PRINCIPLE OF SURGICAL PRACTICE.*

BY THOMAS BRYANT, F.R.C.S.

*From London Lancet, April,* 1875.

I PROPOSE, this evening, to draw your attention to what I believe ought to be a great principle of surgical practice, but to what I fear is not yet sufficiently recognized, or, if so, acted upon; I allude to the principle which, for the sake of pithiness and of practical use, I have called that of "the least sacrifice of parts." Explained more fully the principle is the one that forbids the surgeon to sacrifice more of the body than the absolute necessities of the case demand; that calls upon him to remove the disease that requires removal, but no more; that enables him in accidental surgery to make a flap for an amputation wherever he can, and in some cases to make no flap at all, but to leave the case to nature to repair; and, in pathological surgery, to cut through tissues infiltrated with inflammatory deposits rather than go above a joint, or take away more of a limb than the necessities of the case demand.

To enable me to illustrate this principle fully, I propose, therefore, to bring the subject before you in a series of propositions, each of which I hope to illustrate by cases and clinical remarks.

The propositions I hope to prove are as follows:

1. That in cases of disease or accident no more of the body is to be

* Read in part at the Medical Society of London, January 11th, 1875.

taken away than the necessities of the case demand.

2. That, to carry out this principle, the surgeon may, in *pathological amputations*, fearlessly divide tissues infiltrated with organized inflammatory products, and even cut through the walls of suppurating cavities, or through diseased joints.

3. That, in *accidental surgery*, parts irreparably injured are alone to be removed and no healthy tissues are to be sacrificed in order to perform a recognized and probably a named operation. That to these ends the surgeon ought to utilize even doubtfully viable integument, or even leave a stump to granulate, when, by so doing, some portions of the shaft of a bone can be left, a joint saved, or amputation above a joint avoided.

The first proposition — "That, in cases of disease or accident, no more of the body is to be taken away than the necessities of the case demand"— may at first sight appear a truism; and perhaps some of my hearers may be disposed to think that it is a rule generally followed. Observation, however, has taught me that such a conclusion would be far from correct, and that amputations and excisions of joints are often performed for local disease in cases from which the local disease could otherwise be removed, with far less risk to life, with an equal probability of securing a good result, and with the preservation of a limb or joint.

In the surgery of the foot or hand the truth of these remarks is well exemplified. For let me ask any hospital surgeon if it be not true that—in cases of disease of the metatarsal or tarsal bones and joints—we are too apt to regard any individual case as a good one for a Chopart's amputation, a Pirogoff's, or a Syme's, according to our own fancy or appreciation of the value of one or other of these operations; and if we are not too prone to forget that a good recovery of the foot may ensue on the removal of the diseased bone or bones without any amputation at all? And in support of this assertion I need do no more than quote the able advocate of Syme's amputation, as given in Holmes' system of Surgery (2d ed., vol. v.), where he expresses his mature opinion, after much experience, "that Syme's amputation is calculated to supersede entirely that of Chopart, besides taking the place of amputation of the leg in the majority of cases supposed to demand it." I need hardly say that I entirely dissent from these views of the learned writer. I believe that for local disease alone no form of amputation of the foot should be entertained until less severe measures have been employed and failed; that when amputation is called for, the minimum amount of foot should be taken away; that when a Chopart's amputation will suffice, a Pirogoff's should not be thought of; that when a Pirogoff's is applicable, a Syme's should not be entertained; and that an amputation of the whole foot is never to be undertaken when the disease can be removed by a less severe measure; for I cordially agree with Mr. Hancock when he asked the question, in his college lectures, "If anything can be more unphilosophical than to advocate the sacrifice of any bone or joint of the foot for no other reason than that a particular operation should be performed."

To the surgery of other parts these remarks are applicable; for do we not often see fingers amputated, and even thumbs removed, in cases of injury that, if left to nature, would very often be saved, in order that the surgeon may make a clean amputation? Do we not see joints excised that might be saved by free incisions into the joint, or the removal of necrosed bone from the joint, and amputations performed above a joint or high up a limb in order that good flaps may be made? I do not say that these things are done so commonly now as they were, but I do say that they are done far too frequently, and I believe such errors are committed because surgeons are not sufficiently impressed with the great principle of operative surgery that I have just enunciated—that of "the least sacrifice of parts."

